# Water Content of Plant Tissues: So Simple That Almost Forgotten?

**DOI:** 10.3390/plants12061238

**Published:** 2023-03-08

**Authors:** Gederts Ievinsh

**Affiliations:** Department of Plant Physiology, Faculty of Biology, University of Latvia, 1 Jelgavas Str., LV-1004 Rīga, Latvia; gederts.ievins@lu.lv

**Keywords:** clonal plants, fruits, mineral nutrients, salinity, seeds, succulence, water storage

## Abstract

The aim of the present review was to reconsider basic information about various functional aspects related to plant water content and provide evidence that the usefulness of measuring absolute water content in plant sciences is undervalued. First, general questions about water status in plants as well as methods for determining water content and their associated problems were discussed. After a brief overview of the structural organization of water in plant tissues, attention was paid to the water content of different parts of plants. Looking at the influence of environmental factors on plant water status, the differences caused by air humidity, mineral supply, biotic effects, salinity, and specific life forms (clonal and succulent plants) were analyzed. Finally, it was concluded that the expression of absolute water content on a dry biomass basis makes easily noticeable functional sense, but the physiological meaning and ecological significance of the drastic differences in plant water content need to be further elucidated.

## 1. Introduction

Water is indispensable for the functioning of all biological organisms. In plants, water has several functions in comparison to other organisms, including transport processes and transpiration. The mechanical properties of plants are highly dependent on water and its localization in tissues and cells. From a global perspective, water circulation in plants is an integral part of the natural water cycle, which is vital to the functioning of ecosystems. An analysis of the chemical and physical properties of water, as well as basic information about water uptake, transport, and transpiration in plants is beyond the scope of this review, and readers are encouraged to consult specialized reviews for this purpose [1,2,3,4].

Usually, the broad term “plant water status” is used when quantitatively describing the plant-water relationship. Plant water status has several various interrelated components or functional aspects, each describing different parts of this relationship: water potential, water movement, and water content [5]. In practice, one of the most widely used indices of the plant-water relationship is water potential, a complex parameter describing energy-related aspects of water status [6]. The water potential refers to the ability of water in the system to perform physiological functions and depends on the hydrostatic pressure in particular tissues, cells, or cellular compartments (pressure potential); the amount of dissolved solutes (solute potential); interactions with solid surfaces (matrix potential); and the effects of gravity (gravitation potential). Water movement largely depends on interactions between soil (water availability) and the atmospheric environment (air humidity, wind, etc.) through the soil-plant-atmosphere continuum, which can be particularly characterized by measuring the root pressure or transpiration rate. Within the present review, the focus will be on the third component of plant water status: water content.

Saturation with water is a critical concept in understanding plant water status, as precisely formulated by N.C. Turner: “living cells need to be more or less saturated with water to function normally, but they are usually incomplete in this desirable condition” [7]. To quantify the degree of water insufficiency or unsaturation, the actual water content of tissues is expressed relative to that at full saturation or full turgor, denoted as “relative water content” (RWC). Wide use of RWC has been associated with the fact that this parameter shows a certain coherence with experimentally measured water potential.

Full saturation with water is expected to occur when particular tissues reach a state of full turgor. In practice, during measurement of RWC, this is usually achieved by exposing detached tissues to water and allowing unlimited water uptake until saturation, as shown by weight stabilization. However, no special attention has been paid to the fact that different tissues of various plant species can have different values of absolute water content (in grams per mass unit) even at full saturation. However, some physiological and pathological disorders are associated with plant tissues becoming oversaturated with water, as in the case of hyperhydricity in plant tissue culture or water-soaking in plant pathogenesis.

Several papers in the 1980s compared various methods for measuring plant water status [5,7,8]. These studies most commonly indicated that measurement of absolute water content either on a dry or fresh mass basis was “generally unsatisfactory because neither is stable” [5], pointing to possible diurnal and seasonal changes in both dry mass and water content. Indications of the potentially erroneous nature of absolute water content measurements found in earlier works seem to have been fully accepted, leading to the current situation in which RWC is the only water content parameter usually determined and analyzed in different functional studies with plants. There is no doubt that each analytical method has its limitations, which must be clearly stated in any case. However, the objection that “because the dry weight can change diurnally and/or seasonally, comparisons of water content on a dry weight basis are unsatisfactory” [7] loses any meaning when performing relatively short-term comparative studies where sampling is carried out at the same time of the day. Nevertheless, widely used measurements of RWC have another possible problem because of experimental manipulation with plant materials to determine water content at “full saturation” or “full turgidity.” This problem has been previously discussed [5], and several more recent studies have provided experimental evidence that measurement of RWC can lead to underestimated results. Thus, it has been shown that in situations leading to internal osmotic adjustment, as in the case of both salt-affected and dehydrated plants, excess water is absorbed during the measurement procedure to obtain a “fully turgid state” [9]. As a result, measured RCW values are anomalously low.

Sometimes, dry matter content, as an inverse parameter to tissue water content, has been used as indicator of functional differences between species with fast growth rates vs. species exhibiting nutrient conservation strategies [10] or to characterize yield quality, as in the case of potato production [11]. However, in order to avoid possible differences in leaf water content caused by variations in soil moisture, samples are usually rehydrated Therefore, it appears that leaf dry matter content is derived from and represents an inverse parameter for relative water content, and it is susceptible to the same technical problems as described above for the measurement of RWC.

One of the problems related to absolute water content measurements and the use of the obtained results is the expression of the measurement data in a relative manner, either as a percentage of the amount of water either on a dry mass basis or on a fresh mass basis. It has been previously argued that, because of the extremely high proportion of water in fresh biomass, both types of expression are difficult to relate to any functional concept, as water content differences on a fresh mass basis tend to be extremely low (only a change in a few percentages when the actual water content changes by 30%), while differences on a dry mass basis tend to be extremely high (typical values for herbaceous plants being 500–850%) [5]. However, water content on a dry mass basis can be also expressed in absolute units, as grams of water per gram of dry mass. It can be argued that the visibility of the differences (and functional meaning of the results) increases significantly when water content is expressed in grams of water per gram of dry biomass (DM) compared to expressing it as a percentage of fresh or dry biomass. In practice, for example, changes in leaf water content from 85.22% to 80.43% were evaluated as “slight” while significant, as they corresponded only to a 5.6% decrease [12], but conversion to g H_2_O g^−1^ DM resulted in a decrease from 4.8 to 3.1, or 35.4%. In some studies, the same parameter (water content in grams of water per gram of dry mass) has been designated as “succulence” [13]. Alternatively, the ratio between fresh mass and dry mass, also showing the relative proportion of water, has been designated as “degree of succulence” [14]. In order to compare water content values across plant taxa, different conditions, and various experimental systems, all data were converted to absolute units (g H_2_O g^−1^ DM) in the present review.

The aim of the present review was to reconsider basic information about various functional aspects related to plant water content and provide evidence that the usefulness of absolute water content measurements is undervalued in plant biology experiments. The main emphasis of the review is on herbaceous or semi-shrub species, with woody plants being mentioned for sake of comparison in separate places. However, an analysis of changes in plant water content due to differences in soil moisture is beyond the scope of the present review and specialized reviews should be consulted.

## 2. Structural Organization of Water in Plant Tissues

Water molecules in tissues of all living beings, including plants, can be part of macromolecular structures, thereby forming a network of interfaces with different properties and diverse functional roles that generally determine the structural and functional properties of macromolecules [3]. Interfacial water (“bound” water) has different properties than bulk or “free” water. Bound water has been estimated to represent approximately 30% of the total water content of plants [15]. A significant proportion of water at any particular moment in time can be attributed to the water being transported by the xylem to be transpired through the stomata, or by the phloem to ensure circulation flow through the plant. Both types of transported water enable solute transport between plant parts. In addition, water can be transported through the apoplast and symplast as well as by the transmembrane pathway [16]. While the water potential in all separate water-containing compartments (xylem, cell wall, cytoplasm, and vacuole) at equilibrium is identical, the particular components of the water potential can differ significantly. In particular, the osmotic potential is usually high in both the vacuole and apoplast, but the turgor pressure potential is extremely important in the case of the vacuole [17]. In contrast, the gravitation potential is only relevant in tall trees.

It can be expected that the relative proportion of water aimed to be transpired through the leaves at any particular point is significant, given the fact that the amount of transpired water per gram of synthesized organic matter can be as high as 500 g. However, in reality, due to the high proportion of water mass in the total fresh mass of the plant, relatively fast xylem flow velocity, and high transpiration intensity, transpiration water is only a relatively small proportion of the total water content of the plant organism and can usually be ignored. The results of direct measurements are not widely available, but the amount of water transpired by individual plants of *Eichhornia crassipes* within an hour was calculated to be equal to 0.33–0.58% from the total amount of water in these plants [18]. Consequently, approximately 70% of water in plants can be designated as “utilizable water”.

The mechanical properties of plant organs are affected not only by their chemical composition and structure but also by maintenance of water-dependent turgor and rigidity. In this respect, plant tissues represent hydrostatic materials [19] and their resistance to mechanical stress is highly dependent on their water content [20].

Differences in the strength of water binding have been studied mostly from the point of view of desiccation tolerance of recalcitrant seeds [21,22]. Variations in stem water content in woody plants with respect to their drought tolerance is another relatively frequently assessed aspect of water content studies in plant biology [23]. Mostly methods based on infrared and Raman spectroscopy, isothermal sorption measurement, dielectric relaxation techniques, and nuclear magnetic resonance spectroscopy have been used to study water properties in plants. Many of these techniques are rather non-specific or require complex and bulky equipment [24]. Recent developments in the field of portable hardware for non-destructive measurement of water content by means of nuclear magnetic resonance have opened up new experimental possibilities, allowing for continuous water content measurements in growing leaves and other relatively small parts of intact plants [25].

## 3. Water Content of Different Plant Parts

### 3.1. Water in Leaves

Initial assumptions that “the weight of leaves is largely water and therefore the leaf blade is composed mostly of nothing than water” [26] still seem to be valid, as only a small number of studies have addressed differences in leaf water content among different plants or their changes under the effects of variable environmental conditions. However, more information is available regarding leaf succulence with respect to drought adaptation and in response to salinity, and these aspects will be analyzed further.

In different grass species, leaf water content is positively related to the proportion of the total volume occupied by mesophyll plus epidermal cells in their leaves [27]. In addition, the size of mesophyll cells can also positively affect leaf water content.

Water content per unit of dry matter increases in all vegetative parts with increasing genetically determined plant growth rate, expressed as the relative growth rate [28]. In a study with 24 wild plant species cultivated under controlled conditions, plant species with the lowest relative growth rate (100–120 mg g^−1^ day^−1^) had whole plant water contents of 4.8–5.7 g g^−1^, but the fastest growing plants (relative growth rate > 300 mg g^−1^ day^−1^) had whole plant water contents of 8.1–10.1 g g^−1^ [29]. These differences most likely resulted from higher rates of both mineral ion uptake and water absorption in fast-growing species. When two inbred lines of *Plantago major* with different growth rates were compared, the line with 25% higher growth rate appeared to have higher water contents in both leaves and roots (Table 1) [30].

A similar relationship has also been established for woody species. When 30 Mediterranean woody species with different post-fire regenerative strategies from a coastal shrubland were compared, the leaves of resprouting species appeared to have lower water contents, slower growth rates, and longer leaf lifespans compared to the leaves of species regenerating from seeds [45].

It is reasonable to suggest that light conditions (intensity of photosynthetically active radiation, spectral characteristics, photoperiod) can also have a pronounced impact on water content in addition to developmental and growth effects. A study with the stoloniferous plant *Potentilla reptans*, adapted to high light environments, showed that shading conditions resulted in decreased leaf dry mass, increased water content from 4.95 to 8.52 g H_2_O g^−1^, and petioles grew taller and thinner as a result of the shade avoidance response [46].

### 3.2. Water in Fruits

Similar to other plant products, the quality of fleshy fruits is critically dependent on their water content, affecting both storage and suitability for food processing [47]. The functional aspects of water status in fleshy fruits are largely affected by structural differences in the surface as compared to those in leaves: stomata are nonfunctional if present and the cuticle is highly differentiated but usually more water-permeable [48]. Together with an increase in solute content in developing fruits, more water accumulates, resulting in increased fruit volume [49]. It can be supposed that during growth, increased water content occurs through cellular vacuolization, but additional water is accumulated in the pectin fraction of cell walls. During the early stages of maturation, water content still increases [40]. However, the timing and intensity of changes in fruit water content are highly genotype-dependent. During the final phases of maturation and senescence, loss of cellular integrity occurs due to high activity of polysaccharide-depolymerizing enzymes, leading to fruit softening [50]. This directly results in the loss of water compartmentalization, which affects the mechanical properties of the fruit, basically changing the fruit from being crunchy to juicy. The functional aspects of water transport and accumulation during fruit development have been recently reviewed, and readers are encouraged to seek further details [47,51].

While the chemical composition of fruits is related to their relative growth rate and the climacteric/non-climacteric character of maturation [52], no comparative study involving fruit water content has been performed. Purely intuitively, one would think that the water content of mature fruits would be related to their type. Thus, berries and citrus fruits seem to be fleshier than pomes, but these organoleptic characteristics are affected mostly by chemical composition and structure instead of water content. Examples of water content values in different fruits are given in Table 2 [53,54,55,56]. It is evident that watermelons, melons, strawberries, and citrus fruits have the highest values, but bananas have among the lowest. Especially interesting with respect to water content and storage is the case of fruits of the coconut palm, *Cocos nucifera*, known as coconuts. Being a typical drupe, a coconut has three layers—exocarp, mesocarp, and endocarp—of which the first two layers form husk, but the hollow endocarp contains a multinucleate liquid endosperm, known as coconut water [57]. Coconut water is rich in sugars, minerals, vitamins, amino acids, etc., with an actual water content of approximately 15.2–16.0 g H_2_O g^−1^ DM (Table 1) [34].

### 3.3. Water in Seeds

Highly controlled changes in water content are important during plant generative reproduction. In seeds, changes in water content during development are parts of physiological changes that lead to the formation of mature seeds. Seed moisture content decreases throughout its development, mostly due to a disproportionately larger rate of assimilate accumulation in comparison to that during the seed-filling phase followed by active water loss during the maturation phase [58]. The opposite process occurs during the imbibition of quiescent seeds before germination, but this initially relies entirely on physical processes, while further changes are under tight internal control [59]. The water uptake rate of dry seeds largely depends on the structure and chemical composition of different seed parts [60].

From a practical point of view, the amount of water in seeds or “seed moisture content” is an important indicator of their expected storage life and resilience. Seeds, detached from a plant, have limited means for controlling their internal water content, which largely depends on the relative humidity of the surrounding air. When the air humidity increases (or decreases), the seed water content slowly balances accordingly, reaching so-called “equilibrium moisture content”. It is important to note that seeds of a particular taxon have genotype-specific values of equilibrium moisture content for particular air humidity levels, but they also depend on temperature. Seed chemical composition has a significant effect on equilibrium moisture content, and starch-containing seeds usually have higher water sorption abilities compared to oil-containing seeds [61]. An increase in seed moisture as a result of storage in a humid atmosphere significantly reduces the seed’s viability and preservation of its germination capacity, leading to a shortened expected storage period of seed material [62]. For example, even an increase in relative humidity from 20% to 30% (resulting in increase in seed moisture only from 4.4% to 5.6%) can reduce seed longevity approximately two-fold.

In contrast to the majority of plant species (about 90%) for which reducing the seed moisture content and decreasing the temperature will increase seed resilience and maintain viability (aka orthodox seeds), seeds of some species do not survive dehydration or low temperatures (aka recalcitrant seeds) [62]. Due to these differences, the viability of recalcitrant seeds is best preserved when stored at high relative humidity (98–99%) and positive temperature (7–17 °C for tropical species and 3–5 °C for temperate species).

### 3.4. Water in Vegetative Propagules

Underground storage organs of geophytes, bulbs, tubers, and corms act as vegetative propagation organs, and several crop species with bulbs and tubers are essential food plants. Similar to generative propagules, i.e., seeds, the water content of vegetative propagules, such as tubers and bulbs, changes during development and maturation and has immense practical importance with respect to storage and processing.

The water content of potato tubers, often expressed as an inverse parameter, dry matter content, is an important feature during potato storage as well as further for food processing [11]. For example, a dry matter content above 22% (or water content below 2.55 g g^−1^ DM) is necessary to gain product yield and profitability in potato chip production. There are characteristic biochemical changes during the growth of potato tubers, such as increased starch content at the expense of decreased sugar concentration, and water content decreases during potato tuber filling [41,42]. In mature tubers, water is not uniformly distributed, with lower levels in the outside than in the inside of the tuber and also lower levels at both the apical and stem ends (Table 1) [11]. Similar to genotype-dependent variability in chemical composition, water content also differs among potato cultivars [63]. In addition, agrotechnical measures significantly affect the water content of potato tubers. For example, excessive application of nitrogen fertilizers increased tuber water content, thereby reducing their quality [64].

At harvest, bulbs of onions and garlic have a relatively uniform distribution of water, including in the fleshy outer layers. To increase the shelf life, the water content of the outer layers needs to be significantly decreased, leading to the formation of several desiccated layers, i.e., the peel. As a result, the total water content decreases, for example, from 5.09 to 4.26 g g^−1^ DM while the water content of the outer layers is only 0.3 g g^−1^ DM (Table 1) [31].

During storage, bulbs of onions and garlic lose water through transpiration, which reduces their shelf life and quality. Agrotechnical measures during cultivation affect water loss from onion bulbs during storage. For example, increasing the nitrogen fertilizer rate from 100 to 150 kg ha^−1^ increased the water loss from 36% to 57% during 150 days of bulb storage [65]. The time of harvesting and topping as well as the duration of the drying period after harvesting also significantly affects the water content of bulbs and water loss during storage [66]. Similarly, postharvest practices have significant effects on garlic bulb quality, as indicated by changes in the water content of the peel (Table 1) [33,67].

### 3.5. Water in Vegetables

To facilitate a comparison, the water content of different types of vegetables is given in Table 3 [68,69]. In many cases, there is no doubt that the domestication process of crops has selected for traits associated with increased water content compared to their wild ancestors. Unfortunately, more extensive comparative studies on the functional meaning of differences in water content, especially in relation to storage functions, are not available. However, these results are of key importance in the practical context of vegetable storage, food processing, etc. In general, moisture loss during storage is a critical factor that negatively affects the quality of stored vegetable products. Both high temperature and low air humidity facilitates water loss through evaporation and cuticular transpiration, which are highly genotype-dependent characteristics [70].

### 3.6. Water Storage in Woody Plants

Water storage in trees is a rather specific case, mostly due to significantly different anatomical and physiological features of woody plants in comparison to herbaceous species. Water is stored mainly in xylem conduits and extracellular spaces of living vascular tissues (aka elastic water), but capillary water can also be stored in highly lignified or dead xylem cells [71,72]. In addition, succulent trees develop fleshy tissues adjacent to sapwood—outer parenchyma layers—that act as a water storage compartment, and parenchymatous pith and cortical tissues also can act as water reservoirs [73]. The anatomical characteristics of woody stems, such as the proportion of dead and living cells, largely affect water availability during events of decreased water potential [74]. Water storage in stems tissues of woody plants acts as a buffer to compensate for variations in leaf transpiration demands [75], but capillary water mostly protects the viability of the cambium [76].

## 4. Effect of Environmental Factors on Water Content

### 4.1. Effect of Air Humidity on Tissue Water Content

“Hyperhydricity” (formerly known as “vitrification”) is a term used to describe a physiological disorder that frequently occurs in plant tissue culture and often leads to a reduction in propagation and significant losses. Hyperhydricity is thought to be caused by high air humidity within cultivation vessels together with other suboptimal conditions, resulting in the glassy and translucent appearance of cultivated tissues [77]. The phenomenon has been recently reviewed in detail, including hyperhydricity-inducing conditions and possible control measures [78]. It was initially proposed that hyperhydric plants contained too much water, thus it was no surprise that the physiological basis for the phenomenon was established to be most likely associated with tissue oversaturation with water at the level of the apoplast [79,80].

Even non-hyperhydric plant tissues have higher water content under conditions of tissue culture than soil-grown plants. Thus, the leaves of *Armeria maritima* contained 8.0 g H_2_O g^−1^ DM under tissue culture conditions during the multiplication phase by shoot explants, but the water content of actively photosynthesizing leaves stabilized at 4.0–4.5 g H_2_O g^−1^ DM after transfer of acclimated plants to soil [81]. In general, limited gas exchange in tissue culture vessels and use of liquid and semi-liquid media lead to increased humidity in the internal environment [82]. In turn, high air humidity results in developmental abnormalities, including poor stomatal function, and leads to reduced transpiration, especially under low light conditions [83]. As unrooted explants have high rates of water uptake driven by negative osmotic potential in cells, this can lead to water oversaturation due to low transpiration rates. Initially, it was supposed that excess water accumulated in cell protoplasts due to high cell wall permeability caused by relatively low amounts of cellulose and lignin [84]. However, the application of methods for visualizing sites of water accumulation supported that extra water was accumulated in intercellular spaces [85]. As a result of cultivation on gelrite or other hyperhydricity-inducing conditions, apoplastic air volumes in cultured plants were occupied by water, causing hypoxic conditions in tissues and initiating a sequence of responses characteristic of oxygen shortage, including oxidative stress [79,80].

Apart from tissue culture, the cultivation of plants in greenhouses or other closed spaces with limited ventilation can result in a buildup of high air humidity that results in different physiological alterations. Among them, high air humidity results in increased water accumulation in shoots, as shown for semi-aquatic species *Ranunculus sceleratus* (Table 1) [39].

### 4.2. Effect of Mineral Nutrition on Water Content

**Figure 1:**
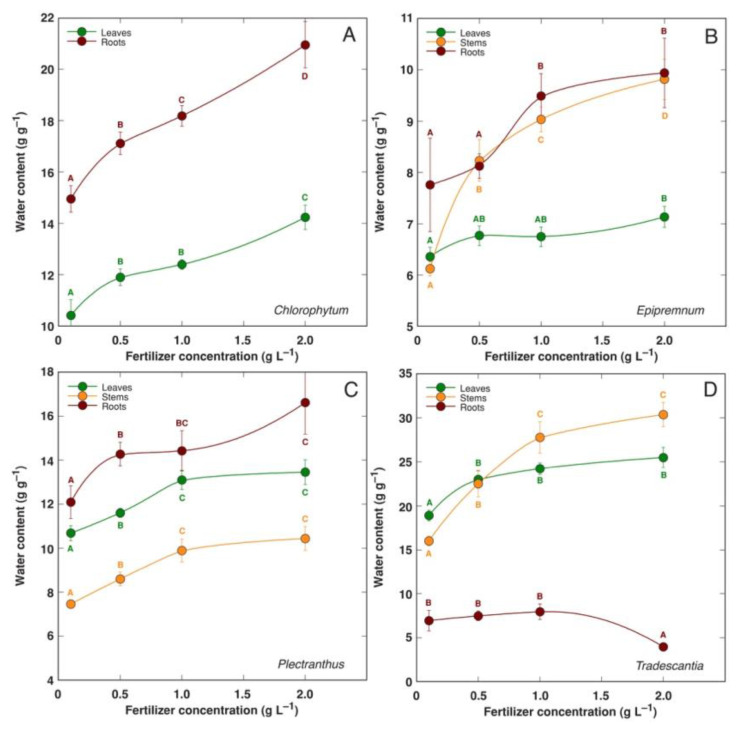
Changes in water content of different parts of hydroponically cultivated *Chlorophytum comosum* (**A**), *Epipremnum aureum* (**B**), *Plectranthus fruticosus* (**C**), and *Tradescantia pallida* (**D**) plants with increasing fertilizer concentration. Different letters indicate statistically significant differences for a particular plant part (*p* < 0.05). Modified from Ievinsh et al., 2021 [43].

An increase in mineral nutrient availability in several species of hydroponically cultivated ornamental indoor plants (*Chlorophytum comosum, Epipremnum aureum, Plectranthus fruticosus, Tradescantia pallida*) resulted in increased water content of tissues (Figure 1) [43]. However, this effect was not evident for extremely slow-growing *Anthurium* spp. and *Spathiphyllum* spp. plants. The water content of both leaves and roots significantly increased with increasing mineral nutrient availability in coastal species *Tripleurospermum maritimum* [86]. The water content of both shoots and roots was increased in soil-grown *Limonium sinuatum* plants with increasing doses of N fertilization, but only up to the N dose that was optimal for plant growth (Table 1) [35].

The water content was increased in shoots of four organically cultivated herb species (*Dracocephalum moldavica, Melissa officinalis, Nepeta cataria, Thymus vulgaris*) in well-watered conditions under the effect of increasing soil amendment with compost and vermicompost, leading to increases in plant-available mineral nutrients in soil, in parallel to increased accumulation of K^+^ and NO_3_^−^ [87]. Although the increased water content of plants by vermicompost amendment has also been noted in other studies [88,89], no clear relationship between increased water content of plant tissues and vermicompost amendment rate has been established. It is also possible that the increased water content is an indirect consequence of improved plant water status due to better water holding capacity of soil after vermicompost amendment [90]. In addition, stimulation of water uptake and accumulation in tissues are known to be the direct effects of application of humic substances, leading to an increase in the fresh mass of plants without much change in dry matter [91].

It seems that the increased water content of plants at luxury mineral nutrient availability is a result of stimulation of vacuolar development as a compartment for ion storage in a similar manner as the process that occurs in salt-adapted plant species at high salinity [92].

### 4.3. Effect of Biotic Interactions on Water Content

Information about the effect of biotic interactions on plant water content is quite difficult to generalize, mainly due to the huge diversity of relatively specific interactions involving plants and other organisms. However, experimental evidence from soil-grown plants under controlled conditions shows that biotic interactions are important determinants of plant water status. This effect is clearly direct in some situations, such as when the organism affecting the plant directly causes changes in the water content of plant tissues, but this effect is indirect in other cases and may rather be related to competition for resources.

One particular case of plant water oversaturation and its role in biotic interactions is associated with microbial pathogens. Many bacterial as well as several fungal pathogens induce the formation of water-soaked lesions on plant leaves during the early phase (~24 h) of infection due to local excessive water accumulation in the apoplast [93]. For *Pseudomonas syringae* pv. *tomato* interaction with *Arabidopsis thaliana*, water soaking is a transient phenomenon that only occurs in compatible interactions [94]. In particular, several bacterial proteins acting as effectors are necessary for the development of an aqueous apoplast environment where aggressive proliferation of bacteria occurs [93].

Plant-plant interactions in soil involve direct competition for resources, both for water and available mineral nutrients. It is highly likely that this also results in changes in plant tissue water content. In addition, plant-parasitic plant interactions represent another situation where a host plant organism acts as a source of resources for a parasitic plant, predominantly either in the form of water, carbohydrates, proteins, and amino acids from the phloem (holoparasites) or water and mineral nutrients from the xylem (hemiparasites) [95]. *Rhinanthus minor*, a facultative root hemiparasite, extracts as much as 20% of the water taken up by the host plant *Hordeum vulgare* [96]. Both water uptake and transpiration dramatically increases in *R. minor* plants after attachment to the host, reflected by the stomata always remaining in an open state, even at night. From the host plant side, the rate of water flow from root to shoot, into leaf sheaths, and into leaf laminae of *H. vulgare* plants after *R. minor* attachment is significantly decreased [97]. Despite a large body of evidence describing different functional aspects of the plant-parasitic plant relationship, changes in the actual water content of plant tissues have rarely been reported. One particular study explored the water regime of unattached non-parasitic vs. host-free attached *R. minor* plants [37]. Contrary to what was expected, the water contents of both shoots and roots of attached *R. minor* plants were even lower than those of unattached plants (Table 1). Thus, it appeared that the increased water flow was used only to maintain a high rate of extraction of host xylem sap for nutrient acquisition instead of increasing the amount of water in tissues. However, similar earlier studies with *Rhinanthus serotinus* and *H. vulgare* indicated that the water content of *R. serotinus* leaves increased after attachment to the host (Table 1) [38]. Surprisingly, usually no effect of parasite attachment on the water status of the host plant has been assessed, besides a highly host genotype-specific effect (from neutral to negative) on the growth of the host plant [98,99]. However, it was shown that water flux from root to shoot also increased in *Nicotiana tabacum* plants infected with the obligate holoparasite *Orobanche cernua* [100].

In contrast to xylem-connecting parasitic plants, where water flow is strong and unidirectional, water flow is bidirectional and weaker in the case of parasitic plants connecting to the host phloem [101]. However, the transpiration rate was increased approximately two-fold in host plants parasitized by *Cuscuta reflexa* in comparison to control plants, together with increased photosynthesis, indicating that the parasite acted as a strong sink [102]. No data on changes in absolute water content are available for *Striga hermonthica*-host interactions, but it has been shown that RWC tended to increase in leaves of *Sorghum bicolor* plants after infection both under wet and dry soil conditions [103]. It appears that, at least for some types of interactions between plants and their parasites, the physiological status of host plants has been excited, allowing for more efficient resource acquisition.

In contrast to the clearly negative effect of parasitic plants on the host plant water regime, there is reason to believe that symbiotic plant interactions, including mycorrhizal symbiosis and nitrogen-fixing bacteria, could have a positive effect in this respect. Indeed, there is a solid body of evidence that mycorrhizal symbiosis affects plant water status under conditions of water shortage [104,105]. Facilitation of water uptake by mycorrhizal hyphae is one of the proposed mechanisms in this respect [106]. A large number of studies have shown the stabilization of RWC in shoots of mycorrhizal plants under drought conditions, in contrast to a decrease in RWC in non-mycorrhizal plants [107,108,109,110,111]. Results on absolute water content are almost absent in this type of study. However, in one study, leaf water content was higher in mycorrhizal *Rosa hybrida* plants under severe water deficit conditions than in non-mycorrhizal plants [112]. A meta-analysis of mycorrhizal effects on plants beyond nutrient acquisition was performed to reveal if arbuscular mycorrhizal fungi had an effect of plant water content (used in a broad sense and including assimilation, leaf water, relative water content, water content, and water use efficiency) [113]. A clearly positive effect of mycorrhiza was found for water flow, mostly due to increased stomatal conductance, but no significant effect was found for water content. It seems that the increase in water potential may be related to increased osmotic potential due to synthesis in osmolytes in mycorrhizal plants [104]. Contradictory results with respect to the beneficial effects of mycorrhiza on plant water status during water shortage can be largely due to genotype-dependent differences as well as variability in experimental conditions. However, in the context of the present review, it is important to understand whether plant water content is affected in mycorrhizal plants under conditions of good water availability. Results of a single study reported that mycorrhizal *Allium porrum* plants had higher water content of leaves but not roots at optimum soil moisture (Table 1) [32].

In the context of improved N availability for legume plants with nitrogen-fixing bacterial symbiosis, it can be expected that water content will be increased in symbiotic plants in comparison to non-symbiotic ones, especially at low soil N concentrations. However, not many experimental results are available to support or deny this hypothesis to date. Based on one study in which both fresh and dry biomass data were available, inoculation of *Trifolium pratense* plants with different *Rhizobium* strains resulted in a decreased water content of both shoots and roots in all combinations (Table 1) [44]. On the other hand, there is evidence that inoculation of *Vigna unguiculata* plants with *Bradyrhizobium* spp. under water shortage conditions improved the drought resistance of plants, but leaf water potential and transpiration rate were not significantly affected by symbiosis [114]. Nevertheless, in a study with drought-stressed *Phaseolus vulgaris*, nodulation with *Rhizobium* spp. partially restored RWC under moderate stress conditions and delayed a decrease in RWC under severe stress conditions [115]. A similar role of nodulation with nitrogen-fixing symbiotic bacteria was also proposed in the case of salinity-affected plants, as rhizobium inoculation partially restored a decrease in RWC in *Medicago truncatula* plants even at high salinity [116].

### 4.4. Water Relationships in Clonal Plants as a Part of Physiological Integration

A relatively large proportion of all known plant species can be designated as clonal or as having complex modularity in the form of potentially autonomous clonal units, i.e., ramets [117]. Clonality in plants is largely an underevaluated phenomenon with immense theoretical and practical importance [118]. Physiological integration in clonal plants involves the division of functions and sharing of resources between ramets, allowing for buffering against environmental heterogeneity. According to the principle of spatial division of functions under conditions of heterogeneous light and water availability, plants will allocate proportionally more biomass to aboveground parts, mostly leaves, of ramets in patches with high light and low water availability and proportionally more biomass in belowground parts of ramets in patches with low light and higher water availability [119]. As a result, clonal species have an advantage with respect to biomass accumulation in more spatially heterogeneous environments. However, particular types of clonal structures are associated with adaptations to habitats with different degrees of heterogeneity: in relatively homogeneous and stable habitats, clonal plants usually exhibit aggregated structure (phalanx morphology) with short spacer distances, but clonal plants with long spacers (guerrilla morphology) usually prevail in highly heterogeneous and dynamic or disturbed habitats [117,120,121].

Features of water transport between ramets within clonal genets dependent on resource availability have been studied. Typically, ramets located in wet patches take up water and transport it to ramets located in dry patches [122]. Importantly, the degree of spatial division of labor depends on the extent of spatial heterogeneity, costs of water transportation, as well as the efficiency of resource capture per unit of biomass [123]. It should be kept in mind that water uptake and transport are the principial mechanisms of mineral nutrient acquisition and distribution [124]. Moreover, water transfer occurs during establishment of new ramets in clonal trees, as in *Populus tremuloides* [125] and bamboo [126]. In particular, bamboo (*Bambusa vulgaris* and *Gigantochloa apus*) plants transfer water from established culms to freshly sprouted culms during their “explosive growth” phase [126].

For clonal plants, especially those belonging to the guerilla-type, ramets show age- and environment-dependent functional specialization associated with differences in their morphology. Thus, older ramets of *Carex bigelowii* have no aboveground structures and are specialized both for uptake of water and minerals as well as for nutrient storage [127]. Resource storage is a functionally important feature of clonal plants, but this aspect has not been particularly studied with respect to water [128].

### 4.5. Succulence as Drought Avoidance

Succulents represent an ecological group of plants that have been studied relatively often with respect to tissue water content. In contrast to the majority of plant species without specialized tissues for water storage, plants with specialized tissues for water storage and having a swollen appearance of stems and leaves are known as “succulents.” Succulence as a functional morphophysiological trait has been attributed either to an ecological strategy of drought avoidance in plants from arid environments or to an ion dilution mechanism in halophytes [92]. Succulence in halophytes will be addressed in the next chapter, with an emphasis here on plants in which water storing tissues act as a reserve water supply for photosynthetic cells during the day to buffer leaf functions against rootzone water shortage. However, even succulent plants with a clear water shortage-avoiding strategy at the cellular level do not need to have the characteristic succulent appearance at the morphological level. Several excellent reviews have summarized both the ecological significance and morphological diversity of succulent plants, functional aspects of their adaptation to the environment, as well as history of knowledge development about succulents [92,129,130,131,132]. According to the aim of the present review, only those properties of succulent plants related to the water content of tissues will be analyzed in more detail.

Many succulents have Crassulacean acid metabolism (CAM)-type photosynthesis where CO_2_ assimilation and photosynthesis are temporally separated, preventing transpiration during the daytime and resulting in higher water use efficiency [133]. However, a causal relationship between the two phenomena is not clear, as CAM species are not the only plants with succulent features [134]. The relationship between leaf succulence and CAM was assessed in 10 species of the genus *Sansevieria* and it was found that presence of CAM was not associated with the amount of leaf hydrenchyma but rather with the manifestation of all cell succulence [135].

The most important feature associated with the morphological diversity of succulents is that water-storing (succulent) tissues can be present in any plant part, as localization in particular tissues has no functional difference with respect to the ability to temporarily ensure independence from external water sources [129]. Similarly, succulence can develop in any tissues of the plant organism, and the resulting tissues can also perform other functions in addition to water storage. Some succulent plant species rely on leaf water storage in expanded chlorenchyma cells without any specialized tissues (all-cell succulents), while other plants have developed achlorophyllous water-storing tissues, known as hydrenchyma (storage succulents) [130]. Spatial organization of storage succulents with respect to mutual arrangement of chlorenchyma and hydrenchyma is extremely variable and the physiological consequences of this variability are far from clear.

Two conceptual functional problems of succulents need to be further solved: whether the amount of stored water is related to drought tolerance and whether the relative proportion of hydrenchyma is related to the amount of stored water.

The concept of “utilizable water” is important part of succulence syndrome, as only a proportion of the total water that constitutes the reserve for maintaining physiological processes during the arid season is taken into account [129]. The localization of water-storing tissues in discrete structures of living cells ensures the maintenance of the water gradient within a plant organism, thus allowing for tight physiological control of water filling and withdrawal. Both the cell size of water-storing tissues and vascular patterning are important determinants for maintaining hydraulic connectivity [134]. However, the relative amount of hydrenchyma was not a significant determinant for water content in *Sanseviera* species, as *Sanseviera parva* and *Sanseviera senegambica* completely lacked hydrenchyma and had relatively higher water contents than species with well-developed hydrenchyma layers (Table 4). In addition, it has been argued that the absolute amount of stored water is not the most important determinant of drought tolerance of succulent plants. During an ecophysiological study of two leaf succulent species from a semi-arid winter rainfall region in Namaqualand (South Africa), deep-rooting C_3_ species *Augea capensis* and flat-rooting CAM species *Malephora purpureo-crocea*, it was established that the water content of both species was higher in winter when more water was available in the soil (Table 4) [136]. However, irrespective of the season, the water content of leaves of the CAM species *M. purpureo-crocea* was 33–44% higher than that of *A. capensis*. In epiphytic species *Pyrrosia lanceolata*, the relative amount of leaf hydrenchyma was positively correlated with the number of hot and rainless days only in the dry season and negatively correlated with the amount of cloud cover in the wet season [137]. However, no obvious link between the degree of succulence and macroclimatic conditions (aridity gradient in native habitats) for five *Crassula* species from southern Africa were found under greenhouse conditions (Table 4) [138].

Rapid uptake and recharge of water in storage tissues is supported by the development of three-dimensional venation system in leaves instead of the common two-dimensional one [139]. Decreased venation density with increased succulence leads to less efficient hydraulic function because of longer distances between veins and photosynthetic tissues. Thus, the three-dimensional venation system is an adaptation that makes it possible to resolve the contradiction between the increased leaf thickness associated with succulence and the need for efficient hydraulic function.

In terms of the spatial organization of water-storing tissues, many leaf and stem succulents are characterized by the presence of specialized parenchymatous cells without chlorophyll, which are localized adjacent to photosynthetic chlorenchyma cells. These enlarged hydrenchyma cells are characterized by a less negative osmotic potential due to a lower concentration of solutes. Water shortage led to a 50% reduction in whole leaf relative water content in *Peperomia magnoliaefolia*, but the hydrenchyma lost 75–85% of water while the chlorenchyma lost only 15–25% of water, effectively protecting the photosynthetic function of the chlorenchyma cells [140]. Initially, under well-watered conditions, the osmotic value of the hydrenchyma was lower than that of the chlorenchyma, mostly due to the higher concentration of sugars in the latter, but the total ion concentration was similar in both cell types. However, during dehydration, osmolality in both tissue types increased to the same extent.

The aerial roots of epiphytic orchids and some other epiphytes from Araceae, such as *Monstera deliciosa*, can absorb water from the atmosphere but the absorbed water is stored in specialized root tissues, i.e., the velamen radicum [141]. These tissues cover the root exodermis and are composed of one or several layers of dead air-filled cells, forming a sponge-like structure. When wetted, velamen cells imbibe water through capillary action. The cell layer located towards the center, the exodermis, has living passage cells within the layer of dead cells, which provide water transport from the velamen to the root cortex where photosynthesizing cells are located. Velamen cells next to the passage cells form special structures, tilosomes, which are particularly densely porous structures that are important for water transport from the velamen to passage cells and further to the cortex. Technically, these plants cannot be classified as succulents because succulence syndrome applies only to plants storing water in living cells. Other epiphytes, such as rootless species from genus *Tillandsia*, have a layer of water-absorptive multicellular trichomes on their leaves for efficient water transport based on capillarity [142].

### 4.6. Effect of Salinity on Water Content

In contrast to succulence syndrome in classical succulent plants, which presumes the presence of morphological adaptations to restrict transpirational water loss, plants with watery leaves with no such adaptations are designated as “fleshy” [129]. However, xerohalophytes, as plants adapted to arid climates, will most likely have morphological adaptations to restrict water loss without having water-storing tissues, whereas hygrohalophytes will have no such characteristics. Both ionic and osmotic relationships are extremely important constituents of plant responses to soil salinity. Both sodium and potassium, the two dominant inorganic-type players, maintain their influence in aqueous medium in the form of ions.

**Figure 2:**
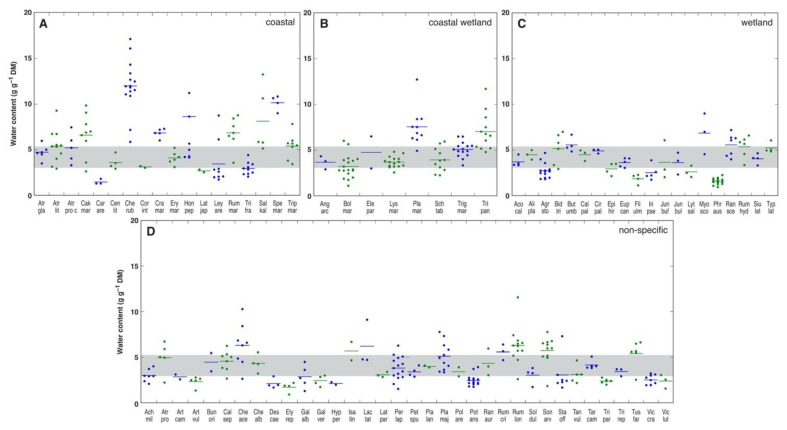
Leaf water content in different species from salt-affected coastal habitats. (**A**), Coastal-specific species; (**B**), coastal-specific wetland species; (**C**), wetland-specific species; (**D**), non-specific species. Each point represents a mean value of water content for a particular distant site. Grey area indicates the middle 50% values among means from all analyzed species. Modified from the data of Ievinsh et al., 2021 [143]. Different color of symbols is used simply for the sake of comprehensibility.

Very little substantial experimental evidence on the effect of salinity on plant water content has been obtained from studies with wild plants in native salt-affected habitats. First, it is clearly understood that any differences in actual water content between samples can be simply due to differences in soil water availability. Second, genotype-, development- and organ-specific variability in water content are highly likely to occur. However, there is reason to believe that plant genotype likely determines salinity-induced changes in tissue water content, as shown in a study with 102 plant species from salt-affected coastal habitats [143]. For each species, water content was measured in the leaves of at least five individuals at each of several geographically distant sites on the coast of the Baltic Sea in Latvia, Estonia, Sweden, and Denmark. In Figure 2, each point represents a mean value of water content for a particular distant site, and it is evident that some species had relatively stable low or high leaf water contents below or above the middle 50% range, respectively. For example, *Carex arenaria* (Car are), *Lathyrus japonicus* (Lat jap), *Filipendula almaria* (Fil ulm), *Phragmites australis* (Phr aus), *Artemisia vulgaris* (Art vul), *Elytrigia repens* (Ely rep), and *Trifolium pratense* (Tri pra) had low leaf water contents, but *Chenopodium rubrum* (Che rub), *Crambe maritima* (Cra mar), *Rumex maritima* (Rum mar), and *Spergularia marina* (Spe mar) had high leaf water contents. Several species showed high variations in leaf water content above the middle 50% range, such as *Honckenya paploides* (Hon pep), *Salsola kali* (Sal kal), *Plantago maritima* (Pla mar), *Tripolium pannonicum* (Tri pan), *Myosotis scorpioides* (Myo sco), *Chenopodium acerifolium* (Che ace), and *Rumex longifolius* (Rum lon). Interestingly, no principle differences emerged between coastal-specific and non-specific or wetland species. Due to clear genotype-specificity in water content in salt-adapted plant species, further mostly experimental evidence from studies performed under controlled conditions will be provided.

“Succulent halophytes” is a descriptive term used for plants that seem to accumulate water under increased soil salinity, presumedly serving both as a water reserve as well as for dilution of tissue salt concentration [144]. However, there are suspicions that the term “succulent” is mostly used to indicate the “fleshy” appearance of leaves and stems. Therefore, two aspects need to be solved in order to define this term from a mechanistic point of view: First, do succulent halophytes possess special water-storing structures? Second, do they accumulate more water under increasing salinity?

Morphologically, a rather unique taxonomic group of halophytes is represented by species of the subfamily Salicornioideae, with Salicornieae (Amaranthaceae/Chanopodiaceae) being the only tribe [145]. The typical visual appearance of these plants includes a fleshy articulate stem with strongly reduced leaves [146]. *Salicornia* and *Sarcocornia* are the two most species-rich genera within the Salicornioideae [145]. Anatomical studies have revealed that the outer layers of the stem are composed of chlorenchymatic photosynthesizing tissues, but the inner layers with peripheral vascular bundles represent chlorophyll-less storage tissues [147]. However, a second cylinder of chlorophyll-containing cells is located around the stele and separated from the water-storing parenchymatous tissues by the layer of endodermis [148]. A physiological division of functions between different layers of stem tissues of *Sarcocornia quinqeflora* has been described, involving the salinity-induced development of the endodermis as a barrier protecting the inner photosynthesizing tissues from salt accumulation [149,150]. For the euhalophyte *Salicornia europaea*, the water content of shoots tended to be higher at moderate salinity, being optimal for growth of the plant, but 800 mM NaCl treatment resulted in a significant decrease in shoot water content (Table 5) [151]. However, this effect was not evident in other studies [152].

The presence of storage tissues has also been described for leaf succulent halophytes. The coastal halophyte *Carpobrotus rossi* (Aizoaceae) has triangular leaves with a narrow outer layer of photosynthetically active mesophyll and inner storage parenchyma cells (80% of leaf volume) surrounding a central vascular strand [173]. The storage parenchyma of salt-affected *C. rossi* plants contains approximately three-fold higher Na^+^ concentrations than mesophyll cells, but the differences in water content between various parts have not been estimated. *Sesuvium portulacastrum* is another halophytic coastal plant species of Aizoaceae [174]. Leaves of *S. portulacastrum* also possess numerous layers of water-storing cells in the center surrounded by 3–7 layers of chlorophyll-containing palisade cells and an outermost layer of epidermis [175]. Tissue culture experiments showed increased water accumulation at optimal salinity but decreased water accumulation at high salinity [170], and a similar response to salinity was reported for intact plants [169].

Another succulent halophyte, *Messembryanthemum crystallinum*, is a C_3_ species able to switch to CAM metabolism under saline, low temperature, or drought conditions [176,177]. The leaf water content of *M. crystallinum* plants growing at optimum salinity was extremely high, reaching 49.0 g H_2_O g^−1^ DM, but it decreased sharply at sea-water salinity (Table 5) [162]. Similarly, in suspension-cultured *M. crystallinum* cells, water content at optimum salinity was 36 g g^−1^ DM [164]. In addition to water storage within leaf tissues, *M. crystallinum* plants use water-filled epidermal bladder cells, representing modified trichomes, to accumulate Na^+^ and other osmotically active substances [178].

In *Atriplex* (*Halimione*) *portulacoides*, the leaf water content increased only at an NaCl concentration stimulating plant growth (200 mM), but leaf dehydration was not evident even at extremely high salinity, causing growth inhibition (Table 5) [156]. Interestingly, at the highest salinity (800 and 1000 mM NaCl) the apoplastic water content in leaves almost doubled, which was associated with maintenance of cell turgidity. For *Atriplex griffithii*, the water content of leaves increased with plant age, but leaf dehydration due to salinity was evident only after 90 days of treatment, with no visible dependence on salinity level [154]. The hygrohalophyte *Atriplex glabriuscula* from coastal drift lines showed no pronounced changes in leaf water content with salinity, but the root water content significantly increased at an NaCl concentration optimal for shoot growth [179]. Xerohalophyte C4 species *Atriplex griffithii* did not show an increase in shoot water content at increased salinity, but leaf dehydration was evident at 500 mM NaCl [180]. Similar results were obtained for another C_4_ xerohalophyte, *Atriplex canescens* [153].

Increased succulence (water content) in leaves of *Suaeda salsa* was associated with increased root hydraulic conductance and induction of expression of an aquaporin-encoding gene, resulting in an increased amount of aquaporin protein in the plasma membrane [14]. However, in another study, increasing salinity (up to 400 mM NaCl) did not result in changes in shoot or root water content in *Suaeda salsa* and *Suaeda glauca*, but the water content of shoots decreased at high concentration of Na_2_CO_3_ (28 mM) in both species [181].

As a result of the previous analysis, it becomes clear that succulent halophytes indeed have specialized water-storing tissues and their water content usually increases with increasing salinity, but only up to a certain salinity level, followed by a decrease in water content. However, it remains to be analyzed what happens to water content under the influence of salinity in relatively salt-tolerant species that usually are not classified as “succulent halophytes.”

**Figure 3:**
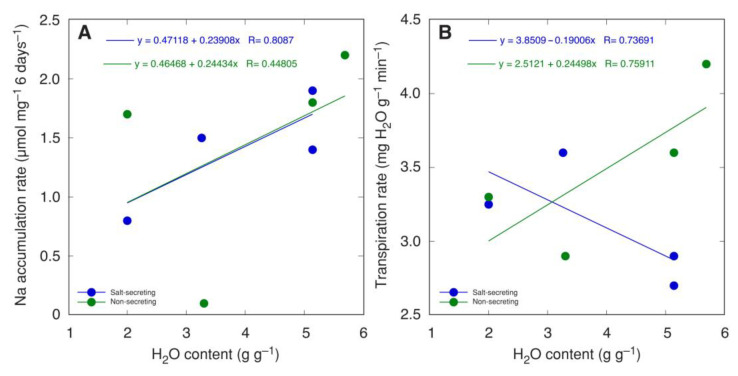
Relationship between shoot water content and Na^+^ accumulation rate (**A**) and transpiration rate (**B**) for salt-secreting (*Glaux maritima, Armeria maritima, Limonium vulgare, Spartina anglica*) and non-salt-secreting (*Juncus maritimus, Juncus articulatus, Atriplex hastata, Atriplex littoralis*) halophyte species grown on 0.2 M NaCl. Data were taken from Rozema et al., 1981 [182] and recalculated.

For a number of salt-secreting (*Glaux maritima, Armeria maritima, Limonium vulgare, Spartina anglica*) and non-salt-secreting halophyte species (*Juncus maritimus, Juncus articulatus, Atriplex hastata, Atriplex littoralis*) grown at moderate salinity, the shoot water content was positively correlated with the Na^+^ accumulation rate (Figure 3A) [182]. However, the relationship between shoot water content and transpiration rate was positive for non-salt-secreting species but negative for salt-secreting species (Figure 3B).

**Figure 4:**
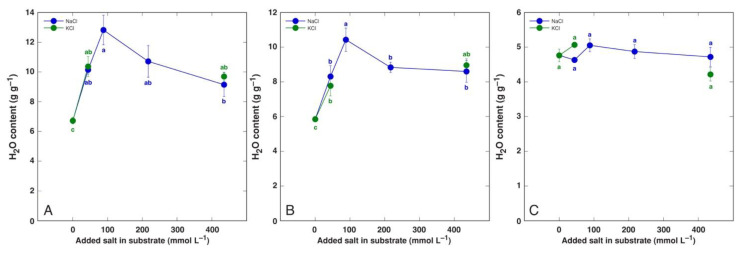
Tissue water content of old leaves (**A**), young leaves (**B**), and roots (**C**) of *Beta vulgaris* subsp. *maritima* plants cultivated in soil and treated with different doses of NaCl and KCl. Different letters indicate statistically significant (*p* < 0.05) differences between treatments. Figure is taken from Ievinsh et al., 2022 [184].

**Figure 5:**
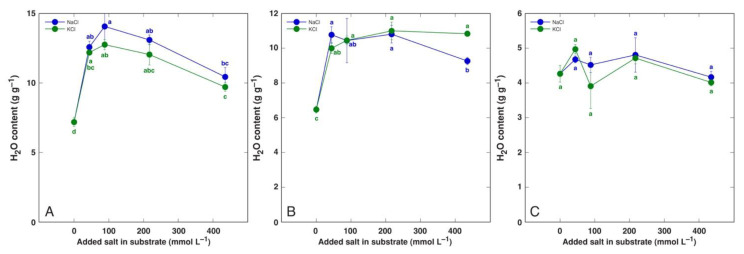
Tissue water content of old leaves (**A**), young leaves (**B**), and roots (**C**) of *Beta vulgaris* var. *cicla* plants cultivated in soil and treated with different doses of NaCl and KCl. Different letters indicate statistically significant (*p* < 0.05) differences between treatments. Figure is taken from Ievinsh et al., 2022 [184].

Species of the genus *Beta* have received special attention in salt tolerance studies due to relatively high salinity tolerance of both wild ancestors as well as cultivated crop forms. The water content of leaves of wild *Beta macrocarpa* plants tended to decrease with increasing salinity, together with a significant increase in apoplastic water content [157]. Even moderate salinity decreased the plant biomass by 40% while increasing the leaf water content in soil-grown leaf beet (*Beta vulgaris* var. *cicla*), but the water content was decreased with increasing salinity [158]. However, in a hydroponic cultivation system, increasing the NaCl concentration up to 100 mM had no significant effect on the leaf water content [183]. In another study, the salinity responses of wild beet ancestor *Beta vulgaris* subsp. *maritima* and cultivated leaf beet crop *Beta vulgaris* var. *cicla* were compared to those of soil-grown plants, and both taxa appeared to be extremely tolerant to both NaCl and KCl salinity, with no growth inhibition up to 400 mmol [184]. Most importantly, the water content of both old and young leaves of both taxa increased, with no significant changes in root water content (Figure 4 and Figure 5).

**Figure 6:**
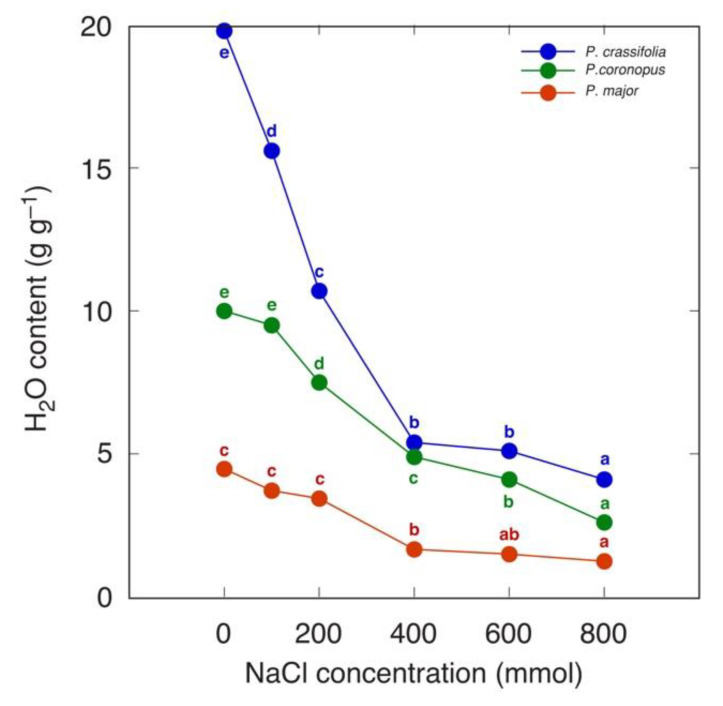
Effect of increasing salinity on leaf water content in three ecologically distinct *Plantago* species. Different letters of respective color indicate statistically significant differences (*p* < 0.05) for the same species. Data were taken from Al Hassan et al., 2016 [185] and recalculated.

**Figure 7:**
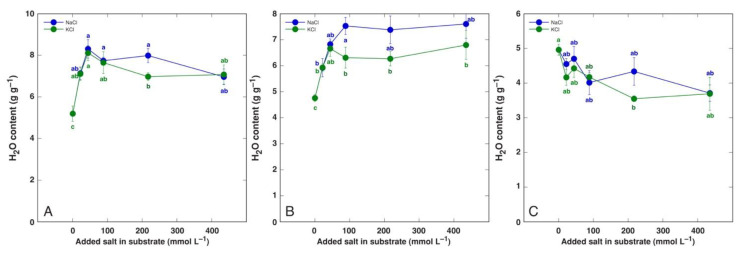
Tissue water content of old leaves (**A**), young leaves (**B**), and roots (**C**) of *Plantago maritima* plants cultivated in soil and treated with different doses of NaCl and KCl. Different letters indicate statistically significant (*p* < 0.05) differences between treatments. Figure is taken from Ievinsh et al., 2022 [184].

Several species of the genus *Plantago* are characteristic of salt-affected habitats and are known as relatively salt tolerant. Different salinity tolerances are reported for various accessions of *Plantago maritima*. An accession from Tunisia was characterized as having relatively low salinity tolerance, with significant growth inhibition already at 50 mM NaCl, and salinity had no stimulative effect on water content (Table 5) [165]. However, the leaf water content was significantly decreased at 200 mM NaCl and decreased linearly with increasing salinity, but the root water content was significantly decreased at 300 mM NaCl. Comparing changes in leaf water content of three *Plantago* species (halophytes *Plantago crassifolia* and *Plantago coronopus*, and salt-sensitive *Plantago major*) differing in salinity tolerance revealed that the rate of leaf dehydration with increasing salinity was relatively similar regardless of particular tolerance level (Figure 6) [185]. Importantly, the leaf water content was more sensitive to increasing salinity than plant development or biomass accumulation. However, for soil-grown *Plantago maritima* plants, the water content of both old and new leaves significantly increased under both NaCl and KCl salinity, with no dehydration evident even at 400 mM salinity, but the root water content tended to decrease with salinity (Figure 7) [184].

**Figure 8:**
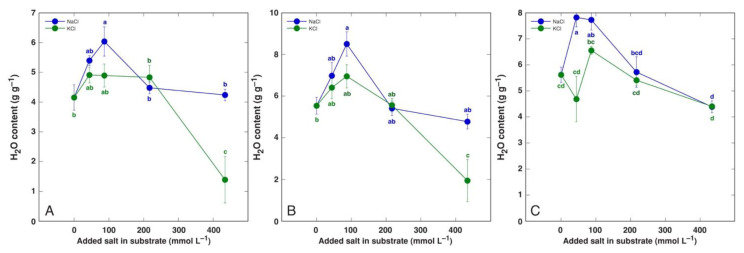
Tissue water content of old leaves (**A**), young leaves (**B**), and roots (**C**) of *Cochlearia officinalis* plants cultivated in soil and treated with different doses of NaCl and KCl. Different letters indicate statistically significant (*p* < 0.05) differences between treatments. Figure is taken from Ievinsh et al., 2022 [184].

**Figure 9:**
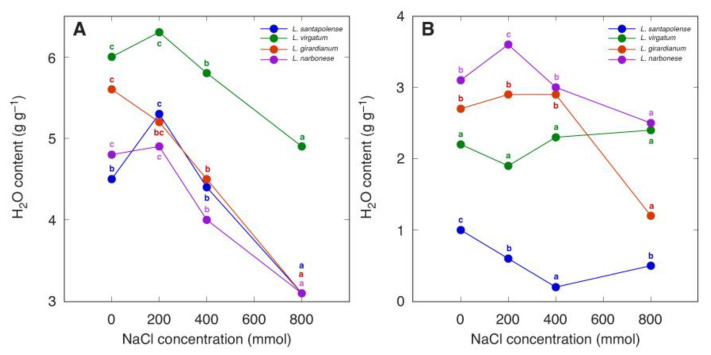
Effect of increasing salinity on water content of leaves (**A**) and roots (**B**) of four *Limonium* species with different geographic distribution patterns. Different letters of respective color indicate statistically significant differences (*p* < 0.05) for the same species. Data were taken from Al Hassan et al., 2017 and recalculated [12].

In leaves of halophytic *Lepidium latifolium* plants, the water content increased by 46% at 300 mM NaCl, but it did not change in the glycophytic species *Lepidium sativum* (Table 5) [159]. Another halophytic species, *Cochlearia officinalis*, showed increased water content of leaf petioles, leaf blades, and roots under moderate NaCl concentrations, with no significant decrease at 400 mM NaCl, but the leaf water content was significantly reduced in plants treated with 400 mM KCl (Figure 8) [184]. For saline wetland species *Tripolium pannonicum* (syn. *Aster tripolium*), the leaf water content was in the range of 5.3–6.6 g g^−1^ DM and was not significantly affected by up to seawater salinity (Table 5) [178]. However, in another study, the water content of both the shoots and roots of *T. pannonicum* decreased at increasing salinity (Table 5) [172]. Similarly, coastal marsh recretohalophyte species *Limonium stocksii* showed decreased water contents of leaves, stems, and roots at a certain level of salinity, but not in a concentration-dependent manner [36]. In other studies with *Limonium sinuatum*, the shoot and root water content was either not affected by salinity [160] or decreased only at high salinity [161]. Four Mediterranean *Limonium* species with different geographical distribution showed extreme tolerance to salinity during early vegetative growth, with significant growth reduction only at 800 mM NaCl and characteristic leaf dehydration already at 600 mM NaCl both in leaves and roots (Figure 9) [12]. Other examples of changes in water content in salt-tolerant species are given in Table 5 [181,182,183,184,185].

Salinity-induced leaf dehydration has been described for several species usually not native to saline habitats, such as *Silene vulgaris* [186] and other species of the genus [187]; *Plantago lanceolata*, *Plantago major*, and *Plantago psyllium* [188]; and *Mentha aquatica* [184]. However, the water contents of leaf petioles, leaf blades, and stems of an extremely salt-tolerant accession of *Ranunculus sceleratus* from a sea water-affected wet beach habitat were also significantly decreased at moderate salinity [39].

**Figure 10:**
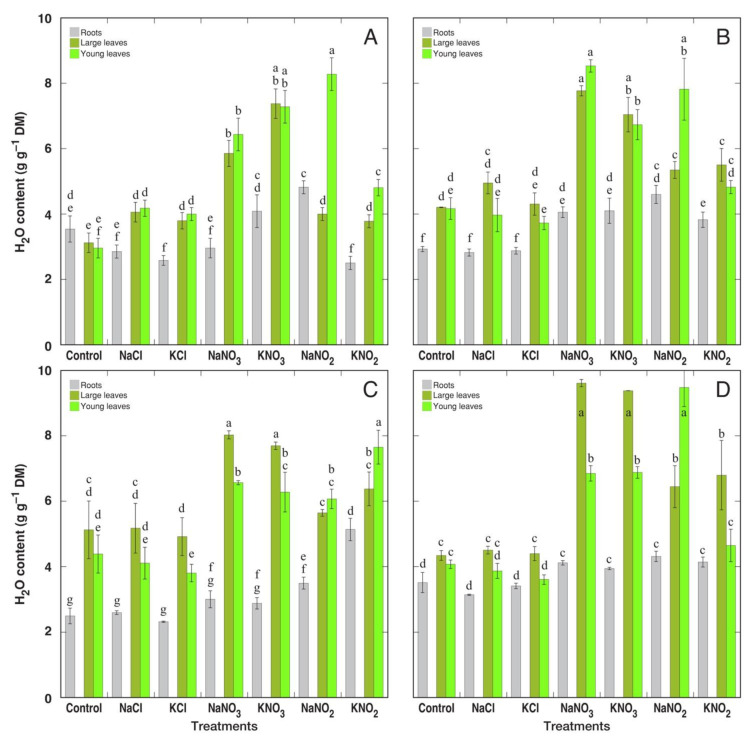
Changes in water content of different parts of *Rumex confertus* (**A**), *Rumex hydrolapathum* (**B**), *Rumex longifolius* (**C**), and *Rumex maritimus* (**D**) plants under the effect of various salinity treatments. Different letters indicate statistically significant (*p* < 0.05) differences between treatments for a particular *Rumex* species. Figure is taken from Ievinsh et al., 2023 [189].

Some of the examples provided above were from studies using both NaCl and KCl as salinity agents, and some differences in water content appeared between the treated plants (Figure 8). However, not only the type of cation but also the type of anion seems to have an effect on salinity-induced changes in plant tissue water content. Thus, for four *Rumex* species, treatment with NaNO_3_ or KNO_3_ resulted in significantly higher water content of leaves in comparison to plants treated with NaCl or KCl (Figure 10) [189]. Moreover, for the three coastal *Rumex* species, *Rumex hydrolapathum, Rumex longifolius*, and *Rumex maritimus*, plants treated with NaNO_2_ or KNO_2_ tended to have higher leaf water content in comparison to treatment with the respective chloride salts, in spite of the fact that the nitrite salts had negative effect on plant growth. At the moment, there is a lack of evidence to make any broader generalizations, but there appears to be some connection between nitrate- and nitrite-stimulated water accumulation and the increased tissue water content described above in the case of an improvement in the supply of fertilizers.

To summarize, some plant taxa do not respond to increasing salinity with changes in tissue water content, at least at moderate salinity, and only mild effects on plant growth are observed, but organ specificity must be taken into account. On the other hand, if the water content changes under the influence of salinity, it can be either an increase or a decrease. As particular studies usually used only a rather limited number of salinity treatments within a particular salinity intensity range, it is difficult to determine a general dose-response pattern; however, it is reasonable to suggest that increased water content appears in species for which a particular salinity level promotes growth, but high salinity causes tissue desiccation in all species, the specific concentration of which will differ depending on the salt tolerance determined by genotype. Indeed, at least in some cases, increased shoot water content seemed to be associated with an increase in dry biomass (growth stimulation by low to moderate salinity), but a causal relationship has not yet been proven. Both types of water changes due to salinity are easily explained from a functional point of view, looking at the increase in water content as an accompanying mechanism for salt accumulation in vacuoles and the decrease in water content (tissue dehydration) as a result of salinity-induced organ senescence and/or decay.

There is no doubt that, similar to variations in salinity tolerance, the response of tissue water content to salinity depends not only on genotype but also on differences in experimental conditions. For salinity tolerance studies, it is indicated that the developmental stage of plants, cultivation system (soil, hydroponics), mineral nutrient availability, light conditions (spectrum, photoperiod, intensity), temperature, mode of salt treatment, etc. are important determinants of morphological and biochemical responses to salinity [190,191,192]. Therefore, it can be expected that responses of tissue water content to salinity may also vary with changes in experimental conditions, and this could explain, at least in part, the conflicting results reported here.

## 5. Conclusions

Available information on the absolute water content of plant tissues and possible functional consequences of the main regularities from both physiological and environmental aspects were analyzed in the present review. The conclusions obtained as a result of the informational analysis can be summarized as follows:

(i) The phenomena of water storage and water content of tissues, apart from drought response studies, has been mostly assessed in the context of succulence syndrome either in classical succulents or succulent halophytes;
(ii) High organ- and species-specific variability can be found with respect to water content in plants;
(iii) The presence of “succulence” and other forms of water storage in particular plant species is not necessarily associated with relatively high water content;
(iv) Salinity can have different effects on water content, even among salt-tolerant and salt-accumulating species;
(v) The expression of absolute water content on a dry biomass basis makes easily noticeable functional sense.

Within the present literature analysis, the highest water content values were evident in the leaves of *Mesembryanthemum crystallinum*, reaching 49 g H_2_O g^−1^ DM at 100 mM salinity, and in the stems and leaves of *Tradescantia pallida*, reaching 30 and 26 g H_2_O g^−1^ DM at high fertilizer concentration, respectively. For the lowest water content values, a number of species had leaf, shoot, or root water contents below 2 g g^−1^ DM under non-saline conditions. However, the fundamental structural and metabolic differences accounting for the drastic differences in plant water content remain unclear. Additionally, above all, it is necessary to clarify the functional meaning and ecological significance of these differences.

## Figures and Tables

**Table 1:** Examples of differences in tissue water content values in various plant species and conditions.

Species	Plant Part	Treatment or Conditions	H_2_O Content (g g^−1^ DM)	Reference
*Allium cepa*	Bulbs	After harvest	5.09	A’yuni et al., 2022 [31]
		After drying, total	4.26	
		After drying, outer layer	0.3	
*Allium porrum*	Leaves	Non-mycorrhizal, 48 h	5.98	Snellgrove et al., 1982 [32]
		Mycorrhizal, 48 h	6.52	
		Non-mycorrhizal, 214 h	4.48	
		Mycorrhizal, 214 h	5.32	
	Roots	Non-mycorrhizal, 48 h	9.00	
		Mycorrhizal, 48 h	8.75	
		Non-mycorrhizal, 214 h	6.89	
		Mycorrhizal, 214 h	5.80	
*Allium sativum*	Peel of bulb	Drying 2 days	3.75	Bayat, Rezvani 2012 [33]
		Drying 9 days	1.15	
		Drying 16 days	0.12	
		Drying 23 days	0.07	
*Cocos nucifera*	Fruit endosperm	6 months	15.2	Santoso et al., 1996 [34]
		12 months	16.0	
*Limonium sinuatum*	Shoots	N 0	2.72	Jain et al., 2018 [35]
		N 10 g m^−2^	3.04	
		N 20 g m^−2^	3.48	
		N 30 g m^−2^	3.19	
	Roots	N 0	1.05	
		N 10 g m^−2^	1.87	
		N 20 g m^−2^	2.02	
		N 30 g m^−2^	1.55	
*Limonium stocksii*	Leaves	Greenhouse conditions	4.3–3.7	Zia et al., 2008 [36]
	Stems		2.6–2.8	
	Roots		1.6–2.3	
*Plantago major*	Leaves	A4 faster growing	9.3	Dijkstra, Lambers 1989 [30]
		W9 slower growing	7.2	
	Roots	A4 faster growing	15.1	
		W9 slower growing	11.2	
*Rhinanthus minor*	Shoot	Non-parasitic	4.82	Jiang et al., 2007 [37]
		Host-free attached	4.05	
	Root	Non-parasitic	8.43	
		Host-free attached	6.67	
*Rhinanthus serotinus*	Leaves	Unattached	4.88	Klaren, van de Dijk 1976 [38]
		Attached	8.52	
*Ranunculus sceleratus*	Leaf petioles	High air humidity	18.4	Prokopoviča, Ievinsh 2023 [39]
		Normal air humidity	16.2	
	Leaf blades	High air humidity	11.3	
		Normal air humidity	10.0	
	Stems	High air humidity	13.7	
		Normal air humidity	11.8	
*Rubus idaeus × Rubus ursinus*	Fruits	Green	3.35	Vicente et al., 2007 [40]
		Red 25%	4.88	
		Red 75%	5.06	
		Red 100%	5.90	
		Purple	5.33	
*Solanum tuberosum*	Tubers	32 days after emergence	5.4	Hajjar et al., 2022 [41]
		46 days after emergence	3.4	
		60 days after emergence	2.6	
*Solanum tuberosum*	Tubers	80 days after planting	4.26 d	Solaiman et al., 2015 [42]
		90 days after planting	3.41 c	
		100 days after planting	2.93 b	
		110 days after planting	2.60 a	
*Solanum tuberosum*	Tubers	Outside	2.4	Pritchard, Scanlon 1997 [11]
		Inside	3.5	
		Apical end	2.8	
		Stem end	3.0	
*Tradescantia pallida*	Leaves	Low fertilizer level	19.1	Ievinsh et al., 2022 [43]
		High fertilizer level	27.0	
	Stems	Low fertilizer level	15.1	
		High fertilizer level	30.1	
*Trifolium pratense*	Shoots	Control	7.50	Matse et al., 2020 [44]
		Inoculated CHB 1120	5.41	
		Inoculated CHB 1121	5.53	
		Inoculated BCRC 14266	6.65	
	Roots	Control	14.71	
		Inoculated CHB 1120	7.75	
		Inoculated CHB 1121	9.80	
		Inoculated BCRC 14266	6.41	

DM, dry mass. In some cases, the results may be relatively inaccurate because they were read from graphs with the closest possible accuracy. If the original results were not in units of mass of water per unit of dry mass, they were converted accordingly.

**Table 2:** Examples of differences in tissue water content values in fruits of various plant species.

Species	Plant Part	H_2_O Content (g g^−1^ DM)	Reference
*Actinidia deliciosa*	Peeled fruit	5.25	Sánchez-Moreno et al., 2006 [53]
*Ananas comosus*	Peeled fruit	5.25	Sánchez-Moreno et al., 2006 [53]
*Ananas comosus*	Pulp Peel	6.63 4.78	Morais et al., 2017 [54]
*Ananas comosus*	Peeled fruit	5.03	Saputri et al., 2022 [55]
*Artocarpus heterophyllus*	Peeled fruit	3.44	Saputri et al., 2022 [55]
*Carica papaya*	Peeled fruit	8.09	Sánchez-Moreno et al., 2006 [53]
*Carica papaya*	Peeled fruit	6.91	Untalan et al., 2015 [56]
*Carica papaya*	Pulp Peel	7.20 6.58	Morais et al., 2017 [54]
*Citrullus lanatus*	Peeled fruit	13.29	Sánchez-Moreno et al., 2006 [53]
*Citrullus lanatus*	Pulp Peel	11.99 12.51	Morais et al., 2017 [54]
*Citrus garndis*	Peeled fruit	11.11	Untalan et al., 2015 [56]
*Citrus × sinnensis*	Peeled fruit	6.69	Sánchez-Moreno et al., 2006 [53]
*Cucumis melo*	Pulp Peel	13.93 11.66	Morais et al., 2017 [54]
*Cucumis melo*	Peeled fruit	11.50	Sánchez-Moreno et al., 2006 [53]
*Fragaria × ananasa*	Whole fruit	10.11	Sánchez-Moreno et al., 2006 [53]
*Malus domestica*	Peeled fruit	6.14	Sánchez-Moreno et al., 2006 [53]
*Mangifera indica*	Peeled fruit	5.25	Sánchez-Moreno et al., 2006 [53]
*Manilkara sapota*	Peeled fruit	2.78	Untalan et al., 2015 [56]
*Musa lacatan*	Peeled fruit	2.65	Untalan et al., 2015 [56]
*Musa* spp.	Peeled fruit	3.00	Sánchez-Moreno et al., 2006 [53]
*Musa* spp.	Pulp Peel	3.04 8.80	Morais et al., 2017 [54]
*Musa* spp.	Peeled fruit	3.00	Saputri et al., 2022 [55]
*Passiflora edulis*	Pulp Peel	7.40 6.19	Morais et al., 2017 [54]
*Persea americana*	Pulp Peel	6.52 1.92	Morais et al., 2017 [54]
*Persea americana*	Peeled fruit	3.76	Sánchez-Moreno et al., 2006 [53]
*Prunus armeniaca*	Peeled fruit	7.33	Sánchez-Moreno et al., 2006 [53]
*Prunus avium*	Peeled fruit	4.00	Sánchez-Moreno et al., 2006 [53]
*Prunus domestica*	Peeled fruit	5.25	Sánchez-Moreno et al., 2006 [53]
*Prunus persica*	Peeled fruit	8.09	Sánchez-Moreno et al., 2006 [53]
*Psidium guajava*	Peeled fruit	4.56	Sánchez-Moreno et al., 2006 [53]
*Pyrus communis*	Peeled fruit	6.14	Sánchez-Moreno et al., 2006 [53]
*Rubus idaeus*	Whole fruit	6.14	Sánchez-Moreno et al., 2006 [53]
*Tamarindus indica*	Peeled fruit	0.50	Untalan et al., 2015 [56]
*Vitis vinifera*	Peeled fruit	4.56	Sánchez-Moreno et al., 2006 [53]

DM, dry mass. In some cases, the results may be relatively inaccurate because they were read from graphs with the closest possible accuracy. If the original results were not in units of mass of water per unit of dry mass, they were converted accordingly.

**Table 3:** Examples of differences in tissue water content values of various vegetables.

Species	Plant Part	H_2_O Content (g g^−1^ DM)	Reference
*Asparagus officinalis*	Shoot	11.85	Duke, Atchley 1986 [68]
*Beta vulgaris*	Leaves	9.99	Duke, Atchley 1986 [68]
*Beta vulgaris*	Root	6.87	Duke, Atchley 1986 [68]
*Beta vulgaris* var. *cicla*	Leaves	10.24	Duke, Atchley 1986 [68]
*Brassica oleracea* var. *acephala*	Leaves	6.87	Duke, Atchley 1986 [68]
*Brassica oleracea* var. *botrytis*	Inflorescence	10.24	Hanif et al., 2006 [69]
*Brassica oleracea* var. *capitata*	Leaves	11.50	Hanif et al., 2006 [69]
*Brassica oleracea* var. *capitata*	Leaves	12.16	Duke, Atchley 1986 [68]
*Brassica rapa* var. *rapa*	Root	10.77	Duke, Atchley 1986 [68]
*Colocasia esculenta*	Root	2.70	Duke, Atchley 1986 [68]
*Dioscorea* spp.	Root	2.77	Duke, Atchley 1986 [68]
*Daucus carota*	Root	7.48	Duke, Atchley 1986 [68]
*Daucus carota*	Root	8.09	Hanif et al., 2006 [69]
*Glycine max*	Shoot	6.30	Duke, Atchley 1986 [68]
*Ipomoea batatas*	Root	2.40	Duke, Atchley 1986 [68]
*Lactuca sativa*	Leaves	15.12	Hanif et al., 2006 [69]
*Pastinaca sativa*	Root	3.79	Duke, Atchley 1986 [68]
*Raphanus sativus*	Root	17.18	Duke, Atchley 1986 [68]
*Raphanus sativus*	Root	12.89	Hanif et al., 2006 [69]
*Spinacia oleracea*	Leaves	9.76	Duke, Atchley 1986 [68]
*Spinacia oleracea*	Leaves	24.00	Hanif et al., 2006 [69]
*Vigna radiata*	Shoot	7.93	Duke, Atchley 1986 [68]

DM, dry mass. If the original results were not in units of mass of water per unit of dry mass, they were converted accordingly.

**Table 4:** Examples of differences in tissue water content values in various succulent plant species.

Species	Conditions	Part, Characteristics	H_2_O Content (g g^−1^ DM)	Reference
*Augea capensis*	Field conditions, winter Field conditions, summer	Leaves	9.8 6.8	Veste, Herpicch 2021 [136]
*Malephora purpureo-crocea*	Field conditions, winter Field conditions, summer	Leaves	13.0 9.8	Veste, Herpicch 2021 [136]
*Crassula brevifolia*	Greenhouse conditions	Leaves	11.66	Fradera-Soler et al., 2021 [138]
*Crassula multicava*	Greenhouse conditions	Leaves	9.59	Fradera-Soler et al., 2021 [138]
*Crassula nudicaulis*	Greenhouse conditions	Leaves	14.72	Fradera-Soler et al., 2021 [138]
*Crassula tecta*	Greenhouse conditions	Leaves	10.78	Fradera-Soler et al., 2021 [138]
*Sansevieria ballyi*	Greenhouse conditions	Leaves, C_3_	12.29	Martin et al., 2019 [135]
*Sansevieria cylindrica*	Greenhouse conditions	Leaves, CAM	11.33	Martin et al., 2019 [135]
*Sansevieria ehrenbergii*	Greenhouse conditions	Leaves, CAM	1.92	Martin et al., 2019 [135]
*Sansevieria fischeri*	Greenhouse conditions	Leaves, CAM	7.52	Martin et al., 2019 [135]
*Sansevieria gracilis*	Greenhouse conditions	Leaves, C_3_	11.33	Martin et al., 2019 [135]
*Sansevieria parva*	Greenhouse conditions	Leaves, CAM	14.67	Martin et al., 2019 [135]
*Sansevieria raffillii*	Greenhouse conditions	Leaves, CAM	10.50	Martin et al., 2019 [135]
*Sansevieria senegambica*	Greenhouse conditions	Leaves, C_3_	13.39	Martin et al., 2019 [135]
*Sansevieria suffruticosa*	Greenhouse conditions	Leaves, CAM	6.00	Martin et al., 2019 [135]
*Sansevieria volkensii*	Greenhouse conditions	Leaves, CAM	16.18	Martin et al., 2019 [135]

DM, dry mass. If the original results were not in units of mass of water per unit of dry mass, they were converted accordingly.

**Table 5:** Effect of salinity on water content of tissues in various plant species.

Species	Characteristic	Part	Salinity	H_2_O Content (g g^−1^ DM)	Reference
*Atriplex canescens*	Xerohalophyte C_4_	Whole plant	0	9.0	Pan et al., 2016 [153]
			100 mM NaCl	9.3	
			200 mM NaCl	6.7	
			400 mM NaCl	6.1	
*Atriplex griffithii*	Halophyte	Leaves	0	8.7	Khan et al., 2000 [154]
			90 mM NaCl	3.7	
			180 mM NaCl	3.7	
			360 mM NaCl	3.7	
*Atriplex halimus*	Xerohalophyte C_4_	Shoot	0	8.0 a	Khedr et al., 2001 [155]
			50 mM NaCl	7.5 a	
			200 mM NaCl	7.5 a	
			500 mM NaCl	5.7 b	
*Atriplex portulacoides*	Euhalophyte	Leaves	0	7.9 a	Benzarti et al., 2014 [156]
			200 mM NaCl	9.9 b	
			400 mM NaCl	7.8 a	
			800 mM NaCl	7.8 a	
			1000 mM NaCl	6.3 a	
*Beta macrocarpa*	Halophyte	Leaves	0	10.2 a	Hamouda et al., 2016 [157]
			100 mM NaCl	8.9 a	
			200 mM NaCl	7.7 a	
*Beta vulgaris* var. *cicla*	Leaf beet crop	Leaves	0	8.0	He et al., 2022 [158]
			0.3% NaCl	9.6	
			0.5% NaCl	6.7	
			0.7% NaCl	6.1	
*Lepidium latifolium*	Halophyte	Leaves	0	5.63	Hajiboland et al., 2020 [159]
			300 mM NaCl	10.53	
*Lepidium sativum*	Glycophyte	Leaves	0	7.95	Hajiboland et al., 2020 [159]
			200 mM NaCl	8.96	
*Limonium sinuatum*	Recretohalophyte	Shoots	0	4.2	Akat, Altunlu 2019 [160]
			50 mM NaCl	3.9	
			100 mM NaCl	3.9	
		Roots	0	4.9	
			50 mM NaCl	4.8	
			100 mM NaCl	5.1	
*Limonium sinuatum* ‘Compindi White’	Recretohalophyte	Shoots	1 dS m^−1^	1.56	Akat et al., 2020 [161]
			5 dS m^−1^	1.89	
			10 dS m^−1^	1.76	
			20 dS m^−1^	1.36	
		Roots	1 dS m^−1^	0.46	
			5 dS m^−1^	0.48	
			10 dS m^−1^	0.43	
			20 dS m^−1^	0.33	
*Limonium sinuatum* ‘Compindi Deep Blue’	Recretohalophyte	Shoots	1 dS m−1	1.55	Akat et al., 2020 [161]
			5 dS m^−1^	1.66	
			10 dS m^−1^	1.83	
			20 dS m^−1^	1.37	
		Roots	1 dS m^−1^	1.14	
			5 dS m^−1^	1.11	
			10 dS m^−11^	0.93	
			20 dS m^−1^	0.43	
*Mesembryanthemum crystallinum*	Succulent C_3_/CAM euhalophyte	Leaves	100 mM NaCl	49.0	He et al., 2022 [162]
			500 mM NaCl	15.7	
*Mesembryanthemum crystallinum*	Succulent C_3_/CAM euhalophyte	Leaves	0	32.3	Agarie et al., 2007 [163]
			100 mM NaCl	39.0	
			400 mM NaCl	21.2	
		Stems	0	21.2	
			100 mM NaCl	32.3	
			400 mM NaCl	19.0	
*Mesembryanthemum crystallinum*	Succulent C_3_/CAM euhalophyte	Cell suspension culture	0	27.0	Tran et al., 2020 [164]
			50 mM NaCl	31.0	
			100 mM NaCl	36.1	
			200 mM NaCl	35.7	
			400 mM NaCl	32.3	
*Plantago maritima*	Hygrohalophyte	Shoots	0	17.2	Sleimi et al., 2015 [165]
			200 mM NaCl	13.9 *	
			500 mM NaCl	7.5 *	
		Roots	0	9.8	
			300 mM NaCl	9.1 *	
			500 mM NaCl	5.3 *	
*Salicornia europaea*	Succulent euhalophyte	Shoots	0	5.7	Cárdenas-Pérez et al., 2022 [152]
			200 mM NaCl	4.1	
			800 mM NaCl	6.3	
		Roots	0	3.7	
			200 mM NaCl	2.5	
			800 mM NaCl	3.0	
*Salicornia europaea*	Succulent euhalophyte	Shoots	0	8.1	Lv et al., 2012 [151]
			200–300 mM	13.3	
			800–1000 mM	3.0	
*Salicornia rubra*	Succulent euhalophyte	Shoots	0	7.9	Khan et al., 2001 [155]
			200 mM NaCl	7.8	
			400 mM NaCl	12.1	
			600 mM NaCl	9.0	
			800 mM NaCl	8.4	
		Roots	0	6.3	
			200 mM NaCl	2.3	
			400 mM NaCl	6.4	
			600 mM NaCl	15.0	
			800 mM NaCl	8.3	
*Sarcocornia quinqueflora*	Succulent euhalophyte	Roots Leaves	0	1.5	Ahmed et al., 2021 [166]
			400–600 mM	3.0	
			0	9.3	
			600 mM	7.5	
			1000 mM	4.5	
*Sesuvium portulacastrum*	Succulent halophyte	Leaves	0	15.5	Slama et al., 2007 [167]
		Roots	0	12.0	
*Sesuvium portulacastrum*	Succulent halophyte	Leaves	0	11.3	Slama et al., 2008 [168]
			100 mM NaCl	9.1	
*Sesuvium portulacastrum*	Succulent halophyte	Shoots	0	5.88	Rabhi et al., 2012 [169]
			200 mM NaCl	11.28	
			400 mM NaCl	11.80	
		Roots	0	4.26	
			200 mM NaCl	7.63	
			400 mM NaCl	5.55	
*Sesuvium portulacastrum*	Succulent halophyte	Shoots in vitro	0	13.0	Lokhande et al., 2011 [170]
			200 mM NaCl	18.0	
			400 mM NaCl	14.7	
			600 mM NaCl	8.0	
*Suaeda salsa*	Succulent euhalophyte	Leaves	0	11.0	Qi et al., 2009 [14]
			100 mM NaCl	14.0	
*Tripleurospermum maritimum*	Coastal salt-tolerant species	Roots	0	6.2	Ievinsh et al., 2021 [86]
			2 g Na^+^ L^−1^	8.7	
		Leaves	0	3.5	
			2 g Na^+^ L^−1^	3.7	
		Stems	0	2.7	
			2 g Na^+^ L^−1^	3.5	
		Flowers	0	4.0	
			2 g Na^+^ L^−1^	3.9	
*Ranunculus sceleratus*	Coastal salt-tolerant species	Leaf petioles	0	18.4	Prokopoviča, Ievinsh 2023 [39]
			4 g Na^+^ L^−1^	14.7	
		Leaf blades	0	11.3	
			4 g Na^+^ L^−1^	5.1	
		Stems	0	13.7	
			4 g Na^+^ L^−1^	8.0	
*Tripolium pannonicum* (*Aster tripolium*)	Hygrohalophyte	Leaves	0–100% seawater	5.3–6.6	Geissler et al., 2009 [171]
*Tripolium pannonicum* (*Aster tripolium*)	Hygrohalophyte	Shoots	0	6.8	Wiszniewska et al., 2019 [172]
			150 mM NaCl	3.9	
			300 mM NaCl	3.3	
		Roots	0	6.6	
			150 mM NaCl	5.5	
			300 mM NaCl	4.6	

DM, dry mass. In some cases, the results may be relatively inaccurate because they were read from graphs with the closest possible accuracy. If the original results were not in units of mass of water per unit of dry mass, they were converted accordingly. Where originally available, different letters or asterisks indicate statistically significant differences given by the authors of the respective paper.

## Data Availability

All data are taken from published sources.
